# An NGS-Based Phylogeny of Orthotricheae (Orthotrichaceae, Bryophyta) With the Proposal of the New Genus *Rehubryum* From Zealandia

**DOI:** 10.3389/fpls.2022.882960

**Published:** 2022-05-12

**Authors:** Isabel Draper, Tamara Villaverde, Ricardo Garilleti, J. Gordon Burleigh, Stuart F. McDaniel, Vicente Mazimpaka, Juan A. Calleja, Francisco Lara

**Affiliations:** ^1^Centro de Investigación en Biodiversidad y Cambio Global, Universidad Autónoma de Madrid, Madrid, Spain; ^2^Departamento de Biología, Facultad de Ciencias, Universidad Autónoma de Madrid, Madrid, Spain; ^3^Departamento de Biodiversidad, Ecología y Evolución,Universidad Complutense de Madrid, Madrid, Spain; ^4^Departamento de Biología, Geología, Física y Química Inorgánica, Universidad Rey Juan Carlos, Móstoles, Spain; ^5^Departamento de Botánica y Geología, Facultad de Farmacia, Universidad de Valencia, Valencia, Spain; ^6^Department of Biology, University of Florida, Gainesville, FL, United States

**Keywords:** Orthotrichinae, Lewinskyinae, *Ulota bellii*, *Atlantichella*, *Plenogemma*, *Pulvigera*, GoFlag 408 hyb seq

## Abstract

Phylogenomic data increase the possibilities of resolving the evolutionary and systematic relationships among taxa. This is especially valuable in groups with few and homoplasious morphological characters, in which systematic and taxonomical delimitations have been traditionally difficult. Such is the case of several lineages within Bryophyta, like Orthotrichaceae, the second most diverse family of mosses. Members of tribe Orthotricheae are common in temperate and cold regions, as well as in high tropical mountains. In extratropical areas, they represent one of the main components of epiphytic communities, both in dry and oceanic or hyperoceanic conditions. The epiphytic environment is considered a hostile one for plant development, mainly due to its low capacity of moisture retention. Thus, the diversification of the Orthotrichaceae in this environment could be seen as striking. Over the last two decades, great taxonomic and systematic progresses have led to a rearrangement at the generic level in this tribe, providing a new framework to link environment to patterns of diversification. Here, we use nuclear loci targeted with the GoFlag 408 enrichment probe set to generate a well-sampled phylogeny with well-supported suprageneric taxa and increasing the phylogenetic resolution within the two recognized subtribes. Specifically, we show that several genera with *Ulota*-like morphology jointly constitute an independent lineage. Within this lineage, the recently described *Atlantichella* from Macaronesia and Western Europe appears as the sister group of *Ulota bellii* from Zealandia. This latter species is here segregated in the new genus *Rehubryum*. Assessment of the ecological and biogeographical affinities of the species within the phylogenetic framework suggests that niche adaptation (including climate and substrate) may be a key evolutionary driver that shaped the high diversification of Orthotricheae.

## Introduction

During the last decades, the use of molecular phylogenetics has transformed our understanding of biodiversity. Molecular data have provided a powerful tool both to resolve phylogenetic relationships and to delimitate the boundaries of taxa at different taxonomic levels. Whereas much progress has been made using data from Sanger sequencing, Next-Generation Sequencing (NGS) techniques can generate far more data and thus greatly enhance the resolution of the genealogy of life (e.g., Lemmon and Lemmon, 2013; Weitemier et al., 2014; Villaverde et al., 2018; Shah et al., 2021).

One of the most promising NGS methods is target enrichment (Weitemier et al., 2014), which can generate data from hundreds, if not thousands, of low-copy nuclear loci that can be used to reconstruct plant phylogenies (e.g., Liu et al., 2019). Recently, the GoFlag project developed a target enrichment probe set that can be used all along the flagellate land plants (i.e., bryophytes, lycophytes, ferns, and gymnosperms; Breinholt et al., 2021). Data generated from these kits can help resolve backbone relationships within large phylogenies and help elucidate important evolutionary processes such as the diversification of land plants. In addition, they also provide an enormous amount of genetic information that can help to resolve the relationships among closely related species or even among populations (e.g., Villaverde et al., 2018). This is especially valuable in groups with few, and often homoplasious, morphological characters, in which systematic and taxonomic delimitations, have been traditionally difficult and contentious.

Bryophytes, including mosses, liverworts, and hornworts, have been repeatedly considered to be genetically static. For example, Liu et al. (2014) provided evidence of the conservation and stasis of the mitochondrial genome in mosses for over 350 My. Similarly, Rosato et al. (2016) postulated evolutionary rDNA stasis during land colonization and diversification across 480 My of bryophyte evolution, and Dong and Liu (2021) stated that genome structure is also static, especially in mosses. This genetic stability correlates, at least in some cases, with a morphological stasis, as demonstrated by McDaniel and Shaw (2003) for *Pyrrhobryum mnioides* (Hook.) Manuel or Aigoin et al. (2009) for *Hedenasiastrum* Ignatov & Vanderp. Nevertheless, the lack of morphological characters can also be due to recent speciation, since relatively young sister species may have had insufficient time to develop and accumulate phenotypic differences (Renner, 2020). This is especially true in bryophytes, where the low structural complexity of the dominant gametophyte implies fewer taxonomically relevant morphological characters in comparison with other groups of land plants. In such groups, the use of NGS techniques may be especially useful for understanding their relationships.

The mosses are the most diverse bryophyte lineage (Liu et al., 2019) with *ca*. 120 families, and Orthotrichaceae Arn., with an estimated 850 species, is the second most diverse family (Frey and Stech, 2009). Orthotrichaceae comprises two subfamilies, Orthotrichoideae Broth. and Macromitrioideae Broth., which differ in morphological, biogeographic, and ecological traits (Lara et al., 2014). Macromitrioideae is almost exclusively intertropical, whereas Orthotrichoideae is common in temperate and cold regions of both hemispheres, as well as in high tropical mountains. Orthotrichoideae is, in turn, divided into two tribes, Orthotricheae Engler and Zygodonteae Engler (Goffinet and Vitt, 1998; Draper et al., 2021). Of these, Orthotricheae stands out as one of the main components of the epiphytic communities in temperate areas, both in dry (Draper et al., 2006; Lara et al., 2009) and in oceanic or hyperoceanic conditions (Garilleti et al., 2015; Lara et al., 2016). Species within Orthotricheae are acrocarpous mosses, whose gametophores grow erect or rarely decumbent and form cushions or tufts. Their leaves are variously lanceolate, erect or imbricate, sometimes twisted when dry, with upper cells rounded, papillose, and basal cells enlarged, smooth, and with single nerves ending near the leaf apex. Their calyptrae are mitrate, plicate, and commonly hairy, and their sporophytes are either immersed, emergent, or variously exserted, with capsules mostly cylindric, smooth, or commonly furrowed, bearing superficial or immersed stomata, and a double peristome of 16 exostomial teeth alternating with 16 endostomial segments that could be somewhat modified or variably reduced (see, e.g., Lara et al., 2014). In fact, the gametophytes of the different Orthotricheae species are overall morphologically similar, and few species within this group can be identified in the absence of sporophytes. This may be one of the reasons for the numerous taxonomic changes that this group has experienced during the last decades, involving rearrangements affecting the main genera (Goffinet et al., 2004; Plášek et al., 2015; Lara et al., 2016; Draper et al., 2021).

For many years, Orthotricheae was understood to include only two large genera, *Orthotrichum* Hedw. and *Ulota* D.Mohr, but in the last 20 years, these two genera have been, respectively, split into five and three genera (see a revision in Draper et al., 2021). Two phylogenetic reconstructions including a representative selection of the Orthotricheae taxa as currently understood have been published recently (Draper et al., 2021; Wang et al., 2021), based on the analyses of 4 nuclear and chloroplast loci, and 6 loci from all three plant genomes, respectively. These two studies represent an important step forward for the understanding of the possible evolutionary history of this tribe. However, the relationships among some taxa remained unresolved, especially within subtribe Lewinskyinae F.Lara, Garilleti & Draper. Also, both of these studies are based on a limited number of loci and could potentially be misled due to complex evolutionary processes such as incomplete lineage sorting or reticulate evolution that are difficult to resolve with few loci (see, e.g., Degnan and Rosenberg, 2009).

Few attempts have been made to reconstruct the relationships in this group with larger phylogenomic data. In addition to the 6 loci previously mentioned, Wang et al. (2021) analyzed 40 mitochondrial and 82 chloroplast genes for a subset selection of 23 Orthotricheae taxa. These authors provided additional evidence supporting the currently accepted circumscription of genera, but the genus *Atlantichella* F.Lara, Garilleti & Draper was not included. In addition, their sampling included few species from each genus. This sparse sampling is especially notable in the largest three genera: *Orthotrichum* (6 out of ~100 species), *Ulota* (4 out of 70 species), and *Lewinskya* F.Lara, Garilleti & Goffinet (6 out of 70 species). Due to the limited sampling, this study could not address the relationships within Lewinskyinae or discern biogeographic and evolutionary patterns that may help explain the diversification and distribution of Orthotricheae.

In this study, we aim to expand upon recent phylogenetic studies with the analysis of a large-scale nuclear dataset generated using target enrichment and including a wide representation of Orthotricheae species, including the main genera and their subgenera, as traditionally delimited. Specifically, we intend to answer whether the nuclear data support the current delimitation of Orthotricheae at the genus level. As indicated above, this has been tested through the analyses of organellar genomes, but nuclear data have been restricted to the inclusion of *ITS*2 and 26*S* in the phylogenies by Draper et al. (2021) and Wang et al. (2021), respectively. The epiphytic habitat is characterized by its low moisture retention capacity, which is especially harsh in areas with climates including a dry season (e.g., Pugnaire and Valladares, 1999). Consequently, the high diversification of *Orthotrichum*, *Ulota*, and *Lewinskya* in this environment could be seen as striking. As a first step toward explaining the high diversification of Orthotricheae in the hostile epiphytic environment, we assess ecological and biogeographic affinities within the herein generated phylogenetic framework to identify putative drivers of evolutionary success in this group.

## Materials and Methods

### Taxon Sampling

We sampled 80 taxa of Orthotrichoideae, focusing on tribe Orthotricheae, with representatives of 7 of its 9 genera. Details on the samples included in the analyses are shown in Supplementary Table S1, with nomenclature following Tropicos (2022) database and abbreviations of authors of plant names following IPNI (2021) database. Specifically, we included 72 species of Orthotricheae, which constitutes approximately 30% of the accepted species: 26 species (out of 100 accepted) of *Orthotrichum*, 1 (of 2) of *Nyholmiella* Holmen & E.Warncke, 22 (of 70) of *Lewinskya*, 1 (of 4) of *Pulvigera* Plášek, Sawicki & Ochyra, 20 (of 70) of *Ulota*, 1 (of 1) of *Plenogemma* Plášek, Sawicki & Ochyra, and 1 (of 1) of *Atlantichella*.

To root the phylogenetic tree, we used representatives of tribe Zygodonteae as sister group, with 4 species of *Zygodon* Hook. & Taylor and 1 of *Australoria* F.Lara, Garilleti & Draper. As ultimate outgroup, we included 1 species of *Leratia* Broth. & Paris (of the sister subfamily Macromitrioideae).

### DNA Extraction

We extracted DNA from the selected samples with a modified cetyltrimethylammonium bromide (CTAB) protocol (Doyle and Doyle, 1987) described in Breinholt et al. (2021). We used a Geno/Grinder 2010 mill (SPEX CertiPrep, Metuchen, New Jersey, United States) to lyse the cells and performed two rounds of chloroform washes followed by an isopropanol precipitation and an ethanol wash. We added 2 μl of 10 mg/ml RNase A (QIAGEN, Valencia, California, United States) to each sample between chloroform washes to remove RNA contamination.

### Target Enrichment and Sequencing Assembly

We employed a target enrichment approach using the GoFlag 408 probe set to generate a multi-locus nuclear sequence dataset for phylogenetic analyses. The GoFlag 408 probe set targets 408 exons found in 229 single or low-copy nuclear genes and appears to recover many loci across mosses (Breinholt et al., 2021). Library preparation, target enrichment, and sequencing were done by RAPiD Genomics (Gainesville, FL, USA). Protocols for library preparation and hybridization are described in Breinholt et al. (2021). All enriched, pooled libraries were sequenced on an Illumina HiSeq 3,000 platform (Illumina; 2 × 100 bp).

We extracted the targeted loci from the raw sequence reads using a pipeline described in detail in Breinholt et al. (2021), and the scripts and reference sequences are available in Dryad (Breinholt et al., 2020). We trimmed the raw sequence reads with Trim Galore! Version 0.4.4^1^ to remove adapters and bases with a Phred score below 20. We then assembled the targeted loci for each sample using iterative baited assembly (IBA; Breinholt et al., 2018), which conducts a *de novo* assembly with BRIDGER version 2014-12-01 (Chang et al., 2015) based on sequence homology of raw reads to a set of reference sequences for each locus. The IBA seeks to extend the assemblies beyond the target regions and recover as much of the more variable flanking intron regions as possible. In this sense, two types of matrices were generated as: (a) assembled sequences trimmed to the probe region (referred to as Probe Only matrices) and (b) full-length assembled sequences, i.e., including probe regions as well as flaking intron sequences (referred as Full Sequences matrices).

Next, we performed an orthology assessment using the target region sequences based on a tBLASTx (Camacho et al., 2009) search against nine flagellate plant genomes to remove potential paralogs. We also removed possible contaminants by performing a tBLASTx search of the assemblies for each locus against the reference sequences, and we removed any sequences that had the best hit that did not come from a moss. Finally, for each locus, we aligned the recovered sequences from the target regions only (Probe Only) and from the combined target and flanking regions from each locus (Full Sequences) with MAFFT 7.425 (Katoh and Standley, 2013). Putative isoforms from the same taxon were merged with a Perl script that used IUPAC ambiguity codes to represent putative heterozygous sites.

Although this pipeline does not explicitly phase loci, in some cases, a locus alignment might include multiple sequences from some samples, representing cases in which the BRIDGER assembler identified allelic diversity. In these cases, to reduce the possibility of including paralogs in our phylogenetic analyses, for each sample with more than one sequence, we removed all sequences from that sample from the locus alignment. Across the 405 loci from which we recovered sequences from at least four samples, we removed 1934 sequences, while retaining 25,544 single-copy sequences. Also, alignments of the flanking regions often have large gaps with sequences from one or a few samples due to indels and the high variability in the recovered length of the flanking sequence. Thus, to clean the alignment and reduce missing data, we ran a script to remove all columns from the locus alignments with fewer than four nucleotides. Additionally, the Probe Only matrix was also pruned with Gblocks (Castresana, 2000) with the following settings: -t = d -b1 = 51 -b2 = 60 -b3 = 8 -b4 = 8 -b5 = h. Summary statistics were calculated using AMAS (Borowiec, 2016). Finally, individual matrices from both datasets were concatenated into two independent supermatrices (i.e., Full Sequences supermatrix and Probe Only supermatrix).

### Phylogenetic Inference

Trees were inferred using two approaches: (a) a total evidence approach using maximum likelihood (ML) inference based on a concatenated matrix of all loci and (b) a summary species tree method that accounts for the Multiple Species Coalescent (MSC) with ASTRAL-III 5.7.8 (Zhang et al., 2018).

For the total evidence approach, we ran ML analyses of both the Full Sequences supermatrix and the Probe Only supermatrix. Phylogenetic analyses of these two datasets were executed in IQ-TREE 2.0.3 (Minh et al., 2020), after automatic model selection using ModelFinder (Kalyaanamoorthy et al., 2017) with the approximate likelihood ratio test (“-alrt” option). These analyses also included 1,000 bootstrap replicates and 1,000 ultrafast bootstrap (“bb” option). To investigate gene tree versus species tree concordance, we calculated two measures of genealogical concordance in our dataset, the gene concordance factor (gCF) and the site concordance factor (sCF), using the options “-gcf” and “-scf” in IQ-TREE. This approach provides a description of possible disagreement among loci and across sites within the sequence. We considered only branches with ultrafast-bootstrap support values >90% as statistically supported. Trees were plotted in FigTree 1.4.4.^2^

For the summary species tree approach under the MSC, individual gene trees were constructed using RAxML 8.2.12 (Stamatakis, 2014) applying GTR-CAT and 200 bootstrap replicates followed by slow ML optimization with the “-f a” option. Then, branches with BS < 50% were collapsed using Newick utilities (Junier and Zdobnov, 2010). Species tree inference under the MSC approach was then performed using ASTRAL-III, and branch support values were inferred through local posterior probabilities (LPP; Sayyari and Mirarab, 2016). Values of LPP > 0.95 were considered to represent strong branch support, although lower values (LPP = 0.7–0.9) also may indicate high support (Sayyari and Mirarab, 2016). To output quartet support values, we used the “-t 2” option. We plotted pie charts reporting the proportion of quartet values in R (R Core Team, 2020) using the packages ape (Paradis and Schliep, 2019), ggimage (Yu, 2021), ggtree (Yu et al., 2017), treeio (Yu et al., 2017), and their corresponding dependencies.

### Niche Preference Characterization

We categorized the studied species according to their niche preferences (regarding substrate and climate) and distribution. Orthotricheae mosses occur on three types of substrates: rocks, tree trunks (including large branches), and small branches (including twigs). All the species were characterized according to their preferences for one, two, or all three possible substrates, mainly on the basis of the expert knowledge of the authors and always recording the prevailing ecological behavior of the species, without considering the most exceptional situations (Mazimpaka and Lara, 1995). We classified the climatic preferences of each species regarding the degree of humidity of the climatic environment in which the species usually grow. These were also divided into three principal types: arid (with scarce precipitations and long periods of dry season, such as the Mediterranean climate), dry (with scarce to moderate precipitations, but without long periods of dry season), or wet (humid or hyper-humid climates, including local or regional situations with frequent mists that produce horizontal precipitations), and we characterized the species as showing preferences for one, two, or all three of them. Finally, we described the climatic preferences regarding the degree of thermicity according to the latitudinal bands where the species thrive as temperate (including cold-temperate), subtropical, and tropical-montane. Subtropical is used as defined by Troll and Paffen (1964) and includes warm climates, between tropical and temperate, with mean temperatures between 17 and 24°C, as prevail in the Mediterranean basin, southern California, The Cape Region, or Macaronesia. The characterization of the species was completed with a description of their geographical range: Subcosmopolite, Holarctic, Sub-antarctic, Australasia, Europe, Western Europe, Mediterranean basin, Macaronesia, East Africa, South Africa, East Asia, South India, North America, Central America, Caribbean, South America, Tropical Andes, and Patagonia.

## Results

### Capture Success and Data Quality

The target enrichment recovered more than 200 nuclear loci (out of a possible 408) for 71 out of 80 samples, and more than 350 loci for 56 of these samples. Five samples largely failed, recovering 5 or fewer loci (*Lewinskya acuminata*, *L. sordida*, *L. tasmanica*, *O. pilosissimum*, and *O. rivulare*), and consequently, we did not include these samples in our analyses. Only five of the targeted loci were recovered in fewer than 10 samples. The average proportion of missing data in the Probe Only supermatrix was 0.63%, and only 10 out of 408 loci had more than 5% of missing data. The average proportion of parsimony informative sites per locus was 17.6%, and only 22 out of 408 loci had less than 10% of parsimony informative sites. The Full Sequences supermatrix was notably noisier, with an average proportion of missing data of 47%, and only 2 loci with less than 5% of missing data. Summary statistics are available in Supplementary Table S1 (sample statistics including number of loci and percentage of sequencing success) and Supplementary Table S2 (loci statistics including length, number of taxa, and number of variable, parsimony informative and missing data sites for both the Probe Only and the Full Sequences supermatrices). Raw data files are available in the GenBank Sequence Read Archive (SRA) under the BioProject number PRJNA819401. The unique accession number of each sample is available in Supplementary Table S3.

### Phylogenetic Reconstruction

**Figure 1:**
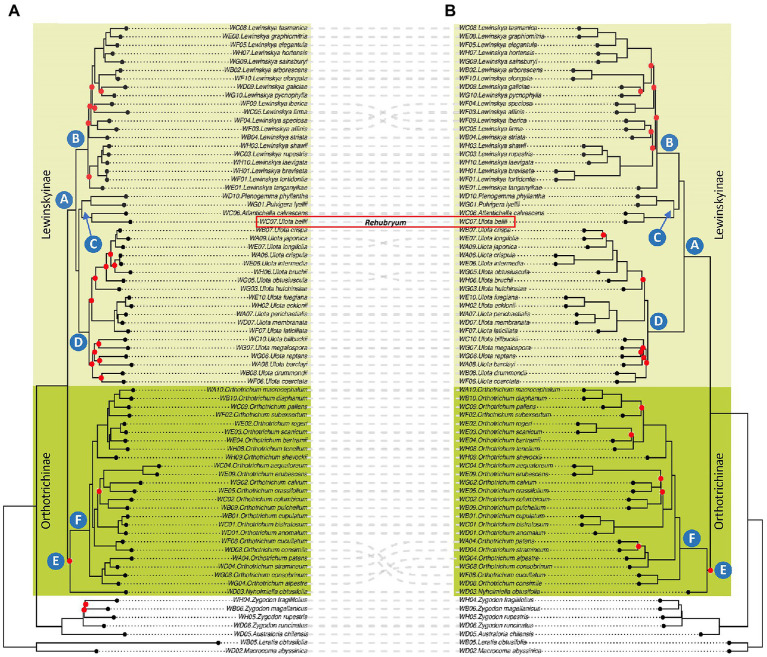
Phylogenetic reconstructions: **(A)** IQ-TREE analysis of the Probe Only supermatrix: concatenation of 21,501 bp after Gblocks pruning. Red dots indicate nodes with ultrafast BS lower than 90. **(B)** ASTRAL analysis of the Probe Only supermatrix: consensus phylogeny based on a total of 405 trees after tBLASTx, with branches with less than 50 BS collapsed. Red dots indicate nodes with LPP lower than 0.90.

The analyses of the Full Sequences supermatrix and the Probe Only supermatrix yielded phylogenetic trees with similar topologies, although the Full Sequences trees showed shorter branches than the Probe Only trees for some of the nodes in the in-group. Therefore, the trees shown on Figure 1 and commented hereafter are those resulting from the Probe Only supermatrix analyses. The trees based on the Full Sequences supermatrix are included as Supplementary Figures, as well as pie charts reporting quartet support values for the Probe Only analyses.

Both species-based trees (IQ-TREE) and gene-based trees (ASTRAL) recover overall concordant clades (Figure 1). Many nodes are well supported with IQ-TREE ultrafastbootstrap (BS; i.e., values greater than 90%) and ASTRAL posterior probability (LPP; i.e., values greater than 0.9). Some of the branches that receive low BS or LPP value support have low gCF scores and/or have low quartet scores (Supplementary Table S4).

Samples of Orthotricheae were included in a single clade sister to samples of Zygdonteae in all the analyses. Within the Orthotricheae clade, samples were distributed in two monophyletic groups, one including the samples of *Orthotrichum* and *Nyholmiella* (not well supported clade E, dark green colored in Figure 1) and the other including the samples from the remaining genera (highly supported clade A, light green). Within clade E, all the analyses placed the sample of *Nyholmiella* as sister to a highly supported clade F, which included all the samples of the species of *Orthotrichum*. This *Orthotrichum* clade F was in turn divided into subclades according to both the species- and gene-based trees, although not all these inner clades were maximally supported, and there was some incongruence on the grouping of *Orthotrichum patens*, *O. stramineum*, *O. alpestre*, *O. consobrinum*, *O. cucullatum*, and *O. consimile*.

Regarding clade A, all the analyses established an inner subgrouping in three large and strongly supported clades, named B, C, and D in Figure 1. The relationships of these three clades varied depending on the analysis: the species-based tree supported a closer relationship between clades C and D, and placed B as sister of the two, whereas the gene-based tree supported a sister relationship for B and C, and placed D as sister of them. Clade B included all the samples of *Lewinskya* and was in turn divided into inner subclades both according to the species- and gene-based trees, although this inner grouping was not maximally supported in any of the analyses. The groups resulting from both the species- and gene-based trees were overall congruent, except for the grouping of *Lewinskya speciosa*, *L. affinis*, *L. iberica*, and *L. firma*. Similarly, clade D was divided into subclades (not fully supported) that were overall congruent among the species- and gene-based trees, except for the relationships established for *Ulota longifolia*, *U. japonica*, *U. bruchii* and *U. hutchinsiae*. This clade D included all the samples of *Ulota* with the exception of the sample of the species *U. bellii*, which was included in clade C together with the small genera *Plenogemma*, *Pulvigera*, and *Atlantichella*.

### Ecological and Biogeographical Affinities

**Figure 2:**
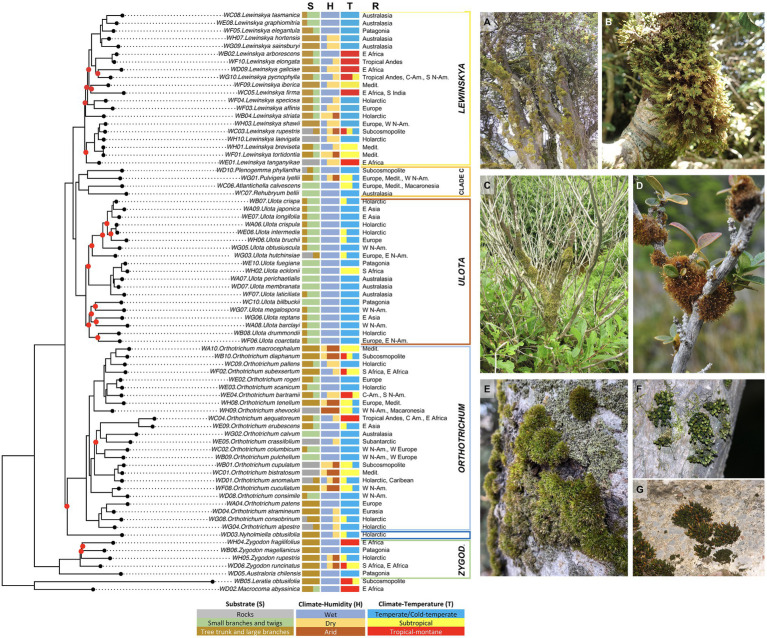
Ecological and biogeographical affinities of the species in the phylogenetic framework (IQ-TREE analysis). For each species, the following information is shown sorted by columns: S—substrate preferences; H—Climate-Humidity, i.e., degree of humidity of the climatic environment in which the species usually grows; T—Climate-Temperature, i.e., degree of climatic thermicity according to the latitudinal climatic bands where the species thrive; R—geographical range. See Materials and Methods section for details on the environmental categories analyzed. Representative pictures: **(A)** *Lewinskya* spp. dominating communities on tree trunks from Bale Mts., Ethiopia; (**B)** *L. graphiomitria* on small branches from Mt. Egmont, North Is., New Zealand; **(C)** *Plenogemma phyllantha* on a shrub trunk and branches from coastal Olympic Peninsula, Washington, United States; and **(D)** *Ulota fuegiana* on shrub twigs from Beagle Channel, Patagonia, Chile. **(E)** *Orthotrichum diaphanum* dominating communities on tree trunk from Madrid, central Spain; **(F)** *O. consobrinum* on a tree trunk from Nara, Honshu Is., Japan; and **(G)** *O. anomalum* on a rock from Burgos, Spain. Abbreviatures: Medit.—Mediterranean Basin, N-Am.—North America; and C-Am.—Central America.

The ecological and biogeographical affinities of the studied species were plotted in the phylogenetic framework to assess whether the recovered clades could reflect ecological or biogeographical patterns. As explained above, the main clades (i.e., clades representing the genus taxonomic level) established by all the analyses performed were congruent, even though the relationships among them sometimes differed. This was the case for the position of clade C, which includes three taxa (*Plenogemma phyllantha*, *Atlantichella calvescens*, and *Ulota bellii*) that have been traditionally treated as *Ulota* due to their morphological similarities. These similitudes were supported by the IQ-TREE analysis, which showed a sister relationship of clade C and *Ulota*. For this reason, we selected the IQ-TREE phylogenetic reconstruction to plot the ecological and biogeographical affinities, in order to identify the possible patterns underlying the recovered clades (Figure 2).

Most of the established clades were clearly congruent with the substrate and climatic preferences. Thus, *Lewinskya* species as a whole tend to be specialized in colonizing tree trunks and large branches, although most of them often also grow on small branches and twigs. Regarding humidity, species of this genus tend to show preferences for dry climate, although some species are typical or common in wet areas, and *L. rupestris*, a cortico-saxicolous species, shows a wide range of humidity tolerance. The highest variability is found among the temperature preferences that range from cold-temperate to tropical-montane at the genus level. Notably, this variability agreed in general terms with the inner subclades of *Lewinskya*, even though these subclades were not always maximally supported. For example, the maximally supported clade including *L. tasmanica* and related species could be defined as typically temperate, whereas the not maximally supported clade of *L. arborescens* and related species could be defined as typically tropical-montane. Conversely, the clades recovered did not show a clear geographical pattern, and species that usually coexist, such as *L. breviseta* and *L. iberica* in the Mediterranean or *L. arborescens* and *L. firma* in East Africa, were not closely related.

The clade including the species of *Ulota* was also clearly differentiated from the rest by its ecological preferences (Figure 2). Regarding substrate affinities, this group of species shows a clear tendency toward small branches and twigs (except for the mainly saxicolous *U. hutchinsiae*), although most of them can also appear on large branches and trunks. As for climatic preferences, these species are all typical of wet areas, temperate, or cold-temperate, from both hemispheres. Exceptionally, they can also thrive in subtropical (Lara et al., 2022) or tropical-montane areas (Garilleti et al., 2015). Again, no clear biogeographical pattern could be established for this genus.

The third large genus of the family is *Orthotrichum*. As shown in Figure 2, this genus is more variable regarding the ecological preferences of its species than the two above mentioned, although some ecological patterns were also obtained, especially for the inner subclades. Thus, this genus includes species usually growing on rocks (some of which were grouped in the subclade including *O. anomalum* and related species), on trunks and large branches (such as those included in the subclade around *O. subexsertum*), and (more rarely) on small branches and twigs. Regarding its climatic preferences, it ranges from an affinity for wet areas to dry or even arid ones and from cold-temperate to tropical-montane areas. No clear pattern was recovered that could easily explain the phylogenetic clades recovered, either based on the climatic or biogeographical affinities.

The remaining five genera of Orthotricheae included in this analysis were represented by a single species each. Four of these were grouped into clade C that, according to the species-based reconstruction, is sister to *Ulota* (Figures 1, 2). This clade C did not represent any biogeographical pattern, since, e.g., *Atlantichella calvescens* and *Ulota bellii*, inferred to be sister taxa in all the analyses, show an antipodal distribution. Conversely, they share a clear preference for small branches and twigs, and climatic preferences for wet areas, from cold-temperate extending to the subtropical latitudes in the case of *A. calvescens*.

## Discussion

### The Use of the GoFlag Enrichment Set in Orthotricheae

The different analyses performed (Probe Only supermatrix *vs* Full Sequences supermatrix; species-based trees *vs* gene-based trees) yielded overall congruent results and well resolved phylogenetic reconstructions. This adds evidence to the utility of the GoFlag enrichment probe set (Breinholt et al., 2021) to resolve not only phylogenetic relationships across distantly related taxa, but also among more closely related taxa, as it has been demonstrated for other groups of flagellate land plants such as ferns (e.g., Fawcett et al., 2021).

Nevertheless, there was discordance regarding the position of some species depending on the analyses (i.e., topology of the ML supermatrix trees *vs* ASTRAL species trees). There are numerous reasons that can explain this type of conflict, including: (i) short branch lengths (i.e., not enough molecular evidence to be able to resolve the phylogenetic relationships among taxa), (ii) incomplete lineage sorting or another type of genuine conflicting genealogical histories (see a revision in, e.g., Degnan and Rosenberg, 2009), or (iii) a selection of loci that are not informative or that provide unclear information (phylogenetic noise, *sensu* Straub et al., 2014). In our case, most of the nodes that produce conflict are associated with short branches, which is especially evident in the case of the inner nodes of *Lewinskya* (clade B in Figure 1) and the position of clade C in relation to clades B and D. The branch suspending the relationship between clade C and clades B-D is very short (due to lack of information, a quick event of diversification, etc.) and thus, some uncertainty involves it: it might appear as sister to either clade B or clade D.

In addition, some of the branches that receive low BS or LPP support have as well low quartet scores, indicating gene tree conflict, and/or have low gCF scores, which reflects that few gene trees support the grouping. For instance, clade E has low BS and LPP support. For this node, which corresponds to a very short branch in both trees, the three quartets have similar values (q1 = 0.39, q2 = 0.29 and q3 = 0.31) and the gCF/sCF are low (14 and 33%, respectively).

In this scenario, it is necessary to further analyze the poorly supported relationships before making conclusions that involve the conflicting nodes within *Lewinskya*, *Ulota*, and *Orthotrichum*. Future studies will include designing a specific target enrichment set for this family, since this could potentially decrease phylogenetic noise and missing data, as it has been previously demonstrated for other groups such as the tribe Cardueae of Compositae (Herrando-Moraira et al., 2019) or the Cyperaceae (Larridon et al., 2020).

### Evolutionary History of the Tribe Orthotricheae

Our phylogenetic reconstruction is the most complete so far published for the tribe Orthotricheae, both in terms of number of taxa included and number of loci analyzed. The results obtained are overall congruent with those published by Draper et al. (2021) and Wang et al. (2021). As proposed by Draper et al. (2021), we confirm that Orthotricheae contains two subtribes, Orthotrichinae F.Lara, Garilleti & Draper and Lewinskyinae, which correspond to clades A (maximally supported) and E (not maximally supported) in Figure 1. Moreover, Draper et al. (2021) proposed the segregation at the genus level of both *Australoria* (separate from *Zygodon*, in Zygodonteae) and *Atlantichella* (separate from *Ulota*), which is also supported by our results (Figure 1).

According to our phylogenetic reconstructions, the genus *Ulota* as currently conceived remains polyphyletic, since *U. bellii* is placed in a separate fully supported clade (namely C, Figure 1) from the rest of the *Ulota* species included in this study (grouped together in a monophyletic and maximally supported clade D in Figure 1). *Ulota bellii* shows a characteristic combination of morphological characters that also justifies its segregation from *Ulota* in a separate genus that we propose to name *Rehubryum* F.Lara, Garilleti & Draper. A brief discussion of the diagnostic morphological characters is provided in the taxonomical description section.

None of the phylogenetic reconstructions so far published has been able to fully resolve the relationships of the different genera within the two subtribes, since many of the clades lacked support. In addition, the relationships suggested by previous studies pointed to incongruent results. Based on their 6-loci results, Wang et al. (2021) suggested that, within Lewinskyinae, *Ulota* is sister to *Lewinskya* and that these are grouped with *Plenogemma* and *Pulvigera* in an unresolved polytomy. Noteworthy, these authors did not include the genera *Atlantichella* and *Rehubryum* in their study. Conversely, Draper et al. (2021) considered *Plenogemma* as sister to *Ulota*, and both of them were grouped in a polytomy with *Lewinskya* and *Atlantichella*, based on a selection of 4 different loci and without representation of *Rehubryum*. Regarding Lewinskyinae, we obtained different topologies depending on the analyses, but all our results point to a sister relationship of *Plenogemma* and *Pulvigera*, as well as of *Atlantichella* and *Rehubryum*, and these four genera are assembled in a monophyletic maximally supported clade C (Figure 1). The sister relationship of this clade, with either *Ulota* (species-based trees) or *Lewinskya* (gene-based trees), remains ambiguous. Our results fail to provide final evidence regarding the phylogenetic relationships for the genera within Orthotrichinae, since we lack data for *Stoneobryum* D.H.Norris & H.Rob. and *Sehnemobryum* Lewinsky & Hedenäs, so further studies are needed to reach final conclusions. Nevertheless, our results point to a different solution than those suggested in the previously published phylogenies and stress the need to further explore this group of taxa to unravel the intergeneric relationships. On one hand, it is necessary to include all the genera of Orthotricheae in a complete phylogeny to resolve the relationships within Orthotrichinae. On the other hand, there is a need to obtain additional molecular data that could help to discern the evolutionary history of the group. As an example, the conflicting solutions suggested by this study (based on nuclear loci) and those previously published (which include data from organellar genomes) could reflect a complex evolutionary history with ancient hybridization events, as it has been observed in other groups such as algae (e.g., Bringloe et al., 2021), angiosperms (e.g., Bogdanova et al., 2021), or other bryophytes (Meleshko et al., 2021).

In any case, our results indicate a puzzling biogeographic history for the extant taxa, given the strongly supported close relationship of *Plenogemma*, *Pulvigera*, *Atlantichella*, and *Rehubryum* shown by all the analyses (clade C, Figure 1). These four taxa include hyperoceanic mosses, but they highly differ in their distributions, reproductive strategies, and morphology (Figure 2). *Pulvigera* comprises four species with *Orthotrichum*-like aspect, all of them found in westernmost North America, although one species can also be found in some Pacific archipelagos, and another one is present in western Europe and the Mediterranean (Lara et al., 2020). All species of *Pulvigera* are dioicous mosses with no specialized vegetative reproduction, except for *P. lyellii*, the one with a disjoint Holarctic distribution, which generates abundant gemmae for vegetative propagation. *Plenogemma phyllantha*, the only representative of its genus, is an *Ulota*-like moss with dioicous distribution of sexes that reproduces mainly by vegetative propagules. It shows a wide and irregular bipolar distribution, involving most continents and several oceanic archipelagoes, including some remote sub-Antarctic islands (Garilleti et al., 2015). In turn, both *Atlantichella* and *Rehubryum* are monotypic genera comprising *Ulota*-like, monoicous mosses that actively reproduce sexually and lack any type of specialized propagules for vegetative reproduction. *Atlantichella calvescens* is an endemic of the northeastern Atlantic area, found in the Macaronesian archipelagoes, British Isles, and scattered localities on the western coast of Europe and the Mediterranean basin (Lara et al., 2022), whereas *Rehubryum bellii* is only known from the Antipodes, restricted to New Zealand.

The close relationship of the four taxa comprising clade C agrees with some morphological similarities (see below) but raises a question about the aspect, sexual system, and distribution of their common ancestors and the evolutionary history of the group. The appearance of the ancestral taxon could be either of the two shown by the current descendants, since an original *Ulota*-like appearance would agree with the topology established by the species tree phylogeny, while the *Orthotrichum*-like appearance would be supported by the topology of the gene-based tree. The two possible sister taxa (*Lewinskya* and *Ulota* s.s.) are entirely monoicous lineages, so the dioicous condition of the subclade formed by *Plenogemma* and *Pulvigera* would in any case be a derived feature, whereas the monoicous condition of the subclade formed by *Atlantichella* and *Rehubryum* would coincide with that of the hypothetic ancestor. We can hypothesize that both the original ancestor and those in the origin of the two main subclades must have been species with high dispersal capacities, as presently shown by many Orthotrichoideae (Vigalondo et al., 2016, 2019). Thanks to recurrent dispersal events, they must have been able to colonize distant hyperoceanic areas of the planet.

In addition to the relationships among the genera of Orthotricheae, this study provides data regarding the infrageneric grouping within the most speciose genera of the tribe. Several infrageneric proposals have been made based on morphological resemblances, all of them focusing exclusively on *Orthotrichum sensu lato* (for a summary, see Lewinsky-Haapasaari and Hedenäs, 1998). Our results suggest that *Lewinskya*, *Ulota*, and *Orthotrichum* can be subdivided into several groups: at least three clades could be recognized within *Lewinskya* (although this grouping is not maximally supported and depends on the analysis performed, species- or gene-based trees); also samples of *Ulota sensu stricto* are distributed in at least three clades, although two of them are not maximally supported; and four groups are established within *Orthotrichum*, all of them maximally supported although their sister relationships partly vary depending on the analyses. Noteworthy, none of these clades is fully congruent with those currently in use (Vitt, 1973; Lewinsky, 1993; Lewinsky-Haapasaari and Hedenas, 1998), although many of the clades here suggested reflect either morphological similarities or ecological preferences (Figure 2). As an example, one of these clades unites the most xerophytic taxa included in the analysis (namely, *O. macrocephalum*, *O. diaphanum*, *O. pallens*, *O. subexsertum*, *O. rogeri*, *O. scanicum*, *O. bartramii*, *O. tenellum*, and *O. shevockii*), while another includes taxa that share stomata located in the lower part of the capsule and a hairy vaginula (*O. patens*, *O. stramineum*, *O. alpestre*, and *O. consobrinum*). Similar results pointing that the traditionally accepted subgenera do not reflect natural phylogenetic groups have been obtained in previous studies (e.g., Goffinet et al., 2004; Sawicki et al., 2012). Unfortunately, this study lacks a complete representation of the diversity of *Orthotrichum* (represented here by 26 of 100 species), *Lewinskya* (22/70), and *Ulota* (20/70), and so the present results are too preliminary as to already propose any new infrageneric division. More data are also needed to increase the resolution of the groups and to be able to infer their taxonomic status.

### Diversification in the Epiphytic Environment

There is a generally accepted assumption that the epiphytic environment constitutes a hostile one for the development of plants, mainly due to drought stress and restricted nutrient supply (e.g., Pugnaire and Valladares, 1999). Nevertheless, it has been also argued that the epiphytic environment can as well be considered as an available space with unexploited resources (Lüttge, 2008) and with a high diversity of microhabitats due to different gradients of light, temperature, humidity, nutrient supply, and substrate characteristics related to bark structure and branch demography (e.g., Zotz, 2016). This has been especially analyzed in tropical forests (e.g., Lüttge, 2008) and on epiphytic vascular plants (Zotz, 2016). A revision synthesizing the underlying biotic interactions that can have been important for epiphyte ecology and evolution has been recently published (Spicer and Woods, 2022). In this study, the authors highlight the importance of acquiring unique adaptive traits to thrive in fine-scale microhabitats within the epiphytic environment, as evolutive drivers in some vascular epiphyte groups. This has been especially claimed for orchids (e.g., Givnish et al., 2015) and bromeliads (e.g., Benzing, 1987), but little has been published on non-vascular plants (Spicer and Woods, 2022) and the specific factors that promote diversification in mosses are not yet well known.

Bryophytes are poikilohydric organisms whose behavior and adaptations to drought stress strongly differ from those of vascular plants (Barkman, 1958). Mosses and other bryophytes compensate the absence of an impermeabilizing epidermis with the ability to enter in a dormancy state that enables them to tolerate desiccation for long periods, whereas water uptake is mostly ectohydric. According to Huttunen et al. (2018), morphological characteristics connected to ectohydry may be driven by adaptations to environmental conditions. This could be interpreted as evidence of how acquiring adaptive traits can drive diversification in bryophytes, which has been suggested for orchids and bromeliads.

Orthotrichaceae is one of the most speciose bryophyte families and has diversified mostly in the epiphytic environment. Within it, the very species-rich subfamily Macromitrioideae has diversified in warm tropical epiphytic environments where also most vascular epiphytes grow. But to what extent the diversification of the tribe Orthotricheae, which specialized in temperate environments (including high tropical altitudes), can be interpreted in the same terms of adaptation to a wide variety of meso- and microenvironmental conditions is something that has not been previously addressed. Previous works lacked support to approach this question, but our results have revealed a clear ecological pattern involving substrate and climatic preferences in the major clades recovered that make progress toward identifying possible drivers of diversification. Conversely, we have not been able to detect a clear biogeographical signal in the phylogenetic reconstructions. This could indicate the evolutionary importance of acquiring adaptative traits that enable the colonization of certain epiphytic microhabitats. In this context, the diversification at the genus level in the tribe Orthotricheae could be partially explained by the adaptation to a certain combination of the substrate characteristics (rocks, trunks, or small branches) together with climatic preferences regarding humidity and temperature. This could also explain an infrageneric diversification, although further data and the inclusion of a wider representation of species are needed to be able to safely achieve conclusions at this taxonomical level. Finally, as suggested by Huttunen et al. (2018) and in line with the results by Draper et al. (2021) on the prevalence of homoplasy among Orthotricheae, the importance of the adaptation to the environment could explain the parallel morphological evolution of the different genera specialized on the epiphytic habitat. Moreover, the results of our study point toward the idea that, at least for bryophytes, stressful environments can promote diversification and harbor great diversity.

### Taxonomical Description

The genus *Rehubryum* is proposed to accommodate *Ulota bellii* Malta on the basis of its peculiar combination of morphological traits and phylogenetic position.

### *Rehubryum* F.Lara, Garilleti & Draper, *gen*. *nov*.

Type: *Rehubryum bellii* (Malta) F.Lara, Garilleti & Draper, comb. nov. ≡ *Ulota bellii* Malta, Acta Horti Bot. Univ. Latv. 7: 15. 1933.

Diagnosis: Plants autoicous, forming cushions. Leaves spirally arranged, strongly crisped when dry, lanceolate, gradually dilated to a base scarcely concave, often plicate on both sides of the nerve, with margins plane or erect-incurved in one side, especially in the transition between base and lamina, leaf lamina unistratose and mainly plane at margins; basal cells long rectangular to linear, somewhat sinuous, with thickened walls; basal-marginal cells differentiated, hyaline, quadrate to rectangular, with thickened transverse walls, forming a narrow marginal band along the base and proximal end of the lamina; margins at upper base with papillose teeth arising at the junctions between two cells; submarginal rows of elongated cells differentiated from base through lower third of the lamina; median and upper leaf-cells rounded to elliptical, with low papillae. Propagula absent. Perichaetial leaves slightly differentiated, with a broader base. Seta 3–5 mm long, twisted counterclockwise. Capsule exserted, oblong-ovoid to short cylindrical, symmetric, entirely ribbed. Exothecial bands narrow differentiated from mouth to urn base. Stomata superficial, at urn base and neck. Peristome double; exostome of 8 pairs of teeth, easily splitting after recurving; endostome of 16 linear segments, involute when dry, with a low connective membrane. Operculum rostrate, with base almost plane. Spores unicellular, isomorphic, papillose. Calyptra mitrate, with abundant stout hairs.

Etymology: *rehu* is a Maori noun for mist but also a verb that means to pass out of sight, disappear, and render unconscious (Moorfield, 2022), all of which seems appropriate for this moss that lives in foggy environments and has gone virtually unnoticed as a different genus.

**Figure 3:**
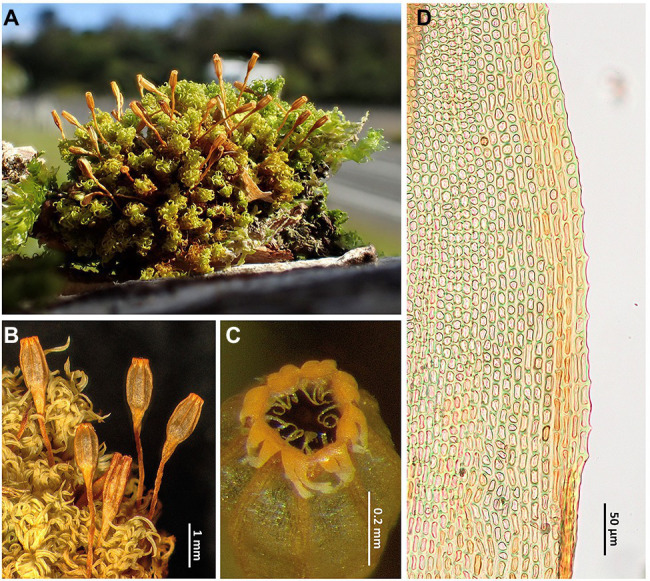
*Rehubryum bellii*. **(A)** General aspect of a dry cushion in the field. **(B)** Detail of the habit showing the upper leaves when dry and several sporophytes, most of them with mature, recently opened capsules. **(C)** Mouth of a capsule when dry, showing a peristome with 8 pairs of teeth, split almost to the base, and 16 well-developed segments. **(D)** Detail of the leaf lamina margin just above the basal leaf showing the submarginal band of elongated cells and the papillose marginal cells. **(A)** image taken in Mount Taranaki NP (Lara 1601/57, MAUAM); **(A–C)** from Garilleti 2016-045b (Garilleti’s personal Herb.); and D from Garilleti 2016–104 (Garilleti’s personal Herb.).

The New Zealand endemic *Rehubryum bellii* appears to be a typical species of the genus *Ulota* as it shows the general look that most of these mosses have (Figures 3A,B), as well as many of the details that serve as morphological characters for their taxonomic characterization. Indeed, in his recent review of *Ulota* in New Zealand, Fife (2017) considers *U. bellii* not worthy of taxonomic recognition and synonymized it with *U. lutea* (Hook. f. & Wilson) Mitt. However, *R. bellii* is easily separated from any species of *Ulota* in the Australasian area by the possession of an endostome consisting of 16 filiform segments, involute when dry, all of them well developed (Figure 3C). Other differential characters, such as the oblong-ovoid shape of the capsule (Figure 3B) or the possession of an exostome with 8 pairs of teeth easily splitting (Figure 3C), have already been highlighted since the description of the species (Malta, 1933; Sainsbury, 1955). However, this moss has two additional very significant characters at the distal part of the leaf base (Figure 3D): (a) submarginal bands of elongate cells ascending from the transition base-blade some way up and (b) margins of some leaves denticulated by prominent papillae arising at the junction between every two marginal cells. Both characters seem to have gone unnoticed and their discovery while examining our New Zealand collections was fundamental for the inclusion of samples of this species in the phylogenetic analysis. In fact, in a previous study (Draper et al., 2021), the occurrence of leaves with submarginal bands of elongate cells was revealed as a characteristic shared by the genera *Atlantichella* and *Plenogemma*, whereas basal leaf margins with geminate teeth are also found in these two genera and in *Pulvigera*, where the feature is especially visible. In contrast, both traits appear to be absent in *Ulota*. The phylogenetic reconstructions obtained in the present study group in the same clade all the four genera in which these features have been so far observed, giving these characteristics an unsuspected taxonomic significance.

The distinction between *Rehubryum bellii* and *Atlantichella calvescens* does not entail any difficulty because there are many morphological differences between the two species, especially in the sporophyte. Thus, for example, whereas *A. calvescens* has capsules broadly ribbed and strongly contracted below mouth when dry, with an endostome of 8 linear segments and devoid of connective membrane, *R. bellii* has capsules finely ribbed, not contracted below mouth when dry, with an endostome of 16 filiform segments and with connective membrane. As these are the only known species of these two genera, it could be thought that their differential traits also serve to characterize *Rehubryum* versus *Atlantichella*. However, all the morphological characters that serve for differentiating both species vary within the large genus *Ulota* (Caparrós et al., 2014; Caparrós, 2015), so their value for characterizing genera among the Lewinskyinae is doubtful. It should also be noted that Draper et al. (2021) demonstrated that within this group of mosses most characters used for separating genera are homoplastic.

## Data Availability Statement

The datasets presented in this study can be found in online repositories. The names of the repositories and accession numbers or links can be found at Figshare: https://figshare.com/s/bb78149766ac8809b4fc; NCBI: PRJNA819401.

## Author Contributions

ID, FL, and RG designed the research. ID, FL, RG, VM, and JC sampled and selected the specimens for the molecular analyses. FL and RG selected and processed the specimens for the morphological study. GB and SM processed the specimens and obtained the sequences. TV, ID, FL, GB, and SM contributed to the phylogenetical analyses. RG, TV, ID, and FL prepared the illustrations. FL, RG, and ID performed the analyses regarding the ecological and geographical preferences. ID and FL wrote a first draft of the manuscript. All authors contributed to the article and approved the submitted version.

## Funding

This research was funded by the Spanish Ministry of Economy, Industry and Competitiveness (grant CGL2016-80772-P), the Spanish Research Agency of the Ministry of Science and Innovation (PID2020-115149GB-C21 and PID2020-115149GB-C22), and the U.S. National Science Foundation (DEB-1541506).

## Conflict of Interest

The authors declare that the research was conducted in the absence of any commercial or financial relationships that could be construed as a potential conflict of interest.

## Publisher’s Note

All claims expressed in this article are solely those of the authors and do not necessarily represent those of their affiliated organizations, or those of the publisher, the editors and the reviewers. Any product that may be evaluated in this article, or claim that may be made by its manufacturer, is not guaranteed or endorsed by the publisher.

## Footnotes

^1^ https://www.bioinformatics.babraham.ac.uk/projects/trim_galore/

^2^ https://github.com/rambaut/figtree/releases
