# Supplementary material for: An NGS-Based Phylogeny of Orthotricheae (Orthotrichaceae, Bryophyta) With the Proposal of the New Genus Rehubryum From Zealandia
Source: Front Plant Sci. 2022 May 12;13:882960. doi: 10.3389/fpls.2022.882960 (PMC9133926; doi:10.3389/fpls.2022.882960)

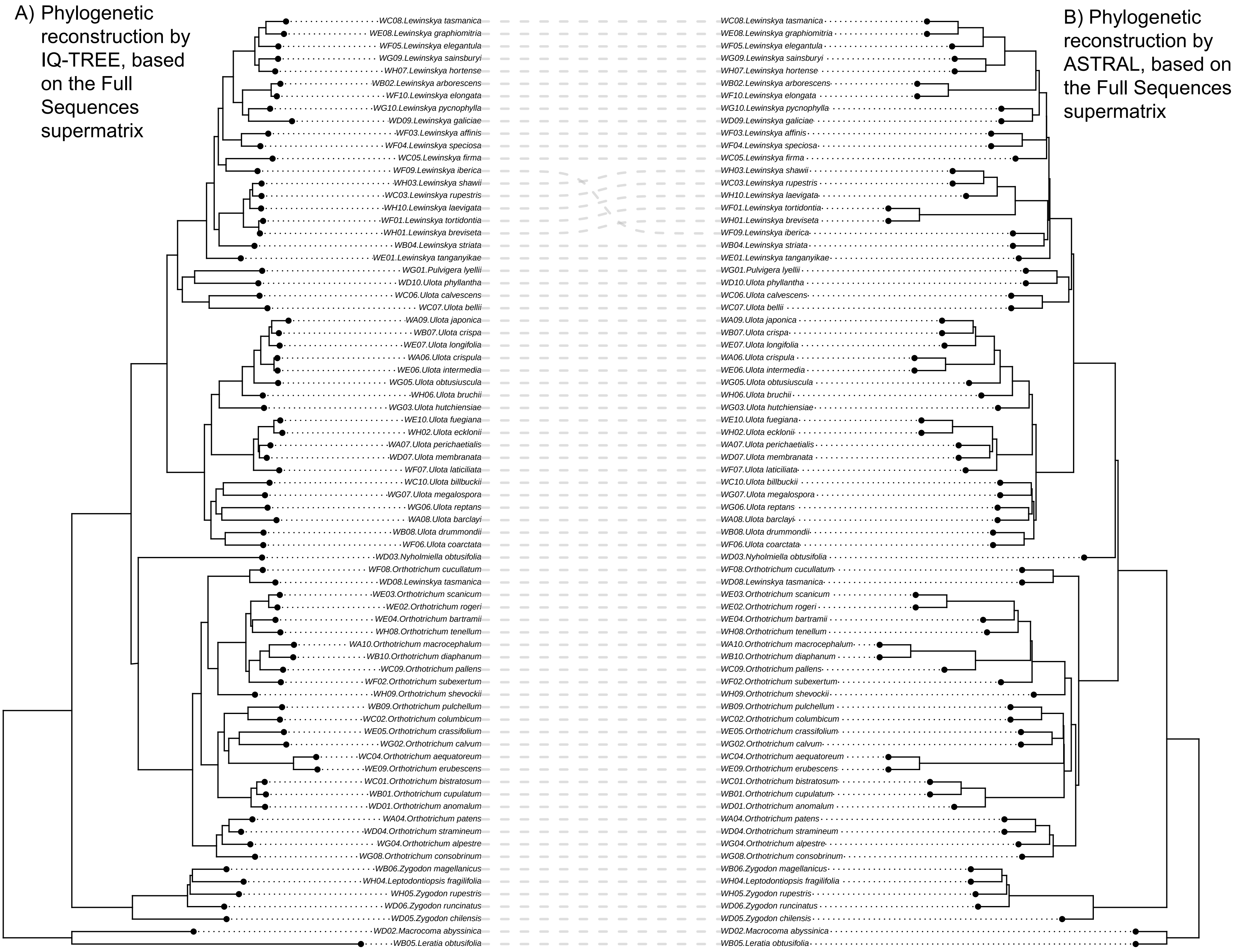

A) Phylogenetic reconstruction by IQ-TREE, based on the Full Sequences supermatrix
WC08.Lewinskya tasmanica
WE08.Lewinskya graphiomitria
WF05.Lewinskya elegantula
WG09.Lewinskya sainsburyi
WH07.Lewinskya hortense
WB02.Lewinskya arborescens
WF10.Lewinskya elongata
WG10.Lewinskya pycnophylla
WD09.Lewinskya galiciae
WF03.Lewinskya affinis
WF04.Lewinskya speciosa
WC05.Lewinskya firma
WF09.Lewinskya iberica
WH03.Lewinskya shawii
WC03.Lewinskya rupestris
WH10.Lewinskya laevigata
WF01.Lewinskya tortidontia
WH01.Lewinskya breviseta
WB04.Lewinskya striata
WE01.Lewinskya tanganyikae
WG01.Pulvigera lyellii
WD10.Ulota phyllantha
WC06.Ulota calvescens
WC07.Ulota bellii
WA09.Ulota japonica
WB07.Ulota crispa
WE07.Ulota longifolia
WA06.Ulota crispula
WE06.Ulota intermedia
WG05.Ulota obtusiuscula
WH06.Ulota bruchii
WG03.Ulota hutchiensiae
WE10.Ulota fuegiana
WH02.Ulota ecklonii
WA07.Ulota perichaetialis
WD07.Ulota membranata
WF07.Ulota laticiliata
WC10.Ulota billbuckii
WG07.Ulota megalospora
WG06.Ulota reptans
WA08.Ulota barclayi
WB08.Ulota drummondii
WF06.Ulota coarctata
WD03.Nyholmiella obtusifolia
WF08.Orthotrichum cucullatum
WD08.Lewinskya tasmanica
WE03.Orthotrichum scanicum
WE02.Orthotrichum rogeri
WE04.Orthotrichum bartramii
WH08.Orthotrichum tenellum
WA10.Orthotrichum macrocephalum
WB10.Orthotrichum diaphanum
WC09.Orthotrichum pallens
WF02.Orthotrichum subexertum
WH09.Orthotrichum shevockii
WB09.Orthotrichum pulchellum
WC02.Orthotrichum columbicum
WE05.Orthotrichum crassifolium
WG02.Orthotrichum calvum
WC04.Orthotrichum aequatoreum
WE09.Orthotrichum erubescens
WC01.Orthotrichum bistratosum
WB01.Orthotrichum cupulatum
WD01.Orthotrichum anomalum
WA04.Orthotrichum patens
WD04.Orthotrichum stramineum
WG04.Orthotrichum alpestre
WG08.Orthotrichum consobrinum
WB06.Zygodon magellanicus
WH04.Leptodontiopsis fragilifolia
WH05.Zygodon rupestris
WD06.Zygodon runcinatus
WD05.Zygodon chilensis
WD02.Macrocoma abyssinica
WB05.Leratia obtusifolia
B) Phylogenetic reconstruction by ASTRAL, based on the Full Sequences supermatrix
WC08.Lewinskya tasmanica
WE08.Lewinskya graphiomitria
WF05.Lewinskya elegantula
WG09.Lewinskya sainsburyi
WH07.Lewinskya hortense
WB02.Lewinskya arborescens
WF10.Lewinskya elongata
WG10.Lewinskya pycnophylla
WD09.Lewinskya galiciae
WF03.Lewinskya affinis
WF04.Lewinskya speciosa
WC05.Lewinskya firma
WH03.Lewinskya shawii
WC03.Lewinskya rupestris
WH10.Lewinskya laevigata
WF01.Lewinskya tortidontia
WH01.Lewinskya breviseta
WF09.Lewinskya iberica
WB04.Lewinskya striata
WE01.Lewinskya tanganyikae
WG01.Pulvigera lyellii
WD10.Ulota phyllantha
WC06.Ulota calvescens
WC07.Ulota bellii
WA09.Ulota japonica
WB07.Ulota crispa
WE07.Ulota longifolia
WA06.Ulota crispula
WE06.Ulota intermedia
WG05.Ulota obtusiuscula
WH06.Ulota bruchii
WG03.Ulota hutchiensiae
WE10.Ulota fuegiana
WH02.Ulota ecklonii
WA07.Ulota perichaetialis
WD07.Ulota membranata
WF07.Ulota laticiliata
WC10.Ulota billbuckii
WG07.Ulota megalospora
WG06.Ulota reptans
WA08.Ulota barclayi
WB08.Ulota drummondii
WF06.Ulota coarctata
WD03.Nyholmiella obtusifolia
WF08.Orthotrichum cucullatum
WD08.Lewinskya tasmanica
WE03.Orthotrichum scanicum
WE02.Orthotrichum rogeri
WE04.Orthotrichum bartramii
WH08.Orthotrichum tenellum
WA10.Orthotrichum macrocephalum
WB10.Orthotrichum diaphanum
WC09.Orthotrichum pallens
WF02.Orthotrichum subexertum
WH09.Orthotrichum shevockii
WB09.Orthotrichum pulchellum
WC02.Orthotrichum columbicum
WE05.Orthotrichum crassifolium
WG02.Orthotrichum calvum
WC04.Orthotrichum aequatoreum
WE09.Orthotrichum erubescens
WC01.Orthotrichum bistratosum
WB01.Orthotrichum cupulatum
WD01.Orthotrichum anomalum
WA04.Orthotrichum patens
WD04.Orthotrichum stramineum
WG04.Orthotrichum alpestre
WG08.Orthotrichum consobrinum
WB06.Zygodon magellanicus
WH04.Leptodontiopsis fragilifolia
WH05.Zygodon rupestris
WD06.Zygodon runcinatus
WD05.Zygodon chilensis
WD02.Macrocoma abyssinica
WB05.Leratia obtusifolia

C) Pie chart reporting quartet support values for the Probe Only analyses

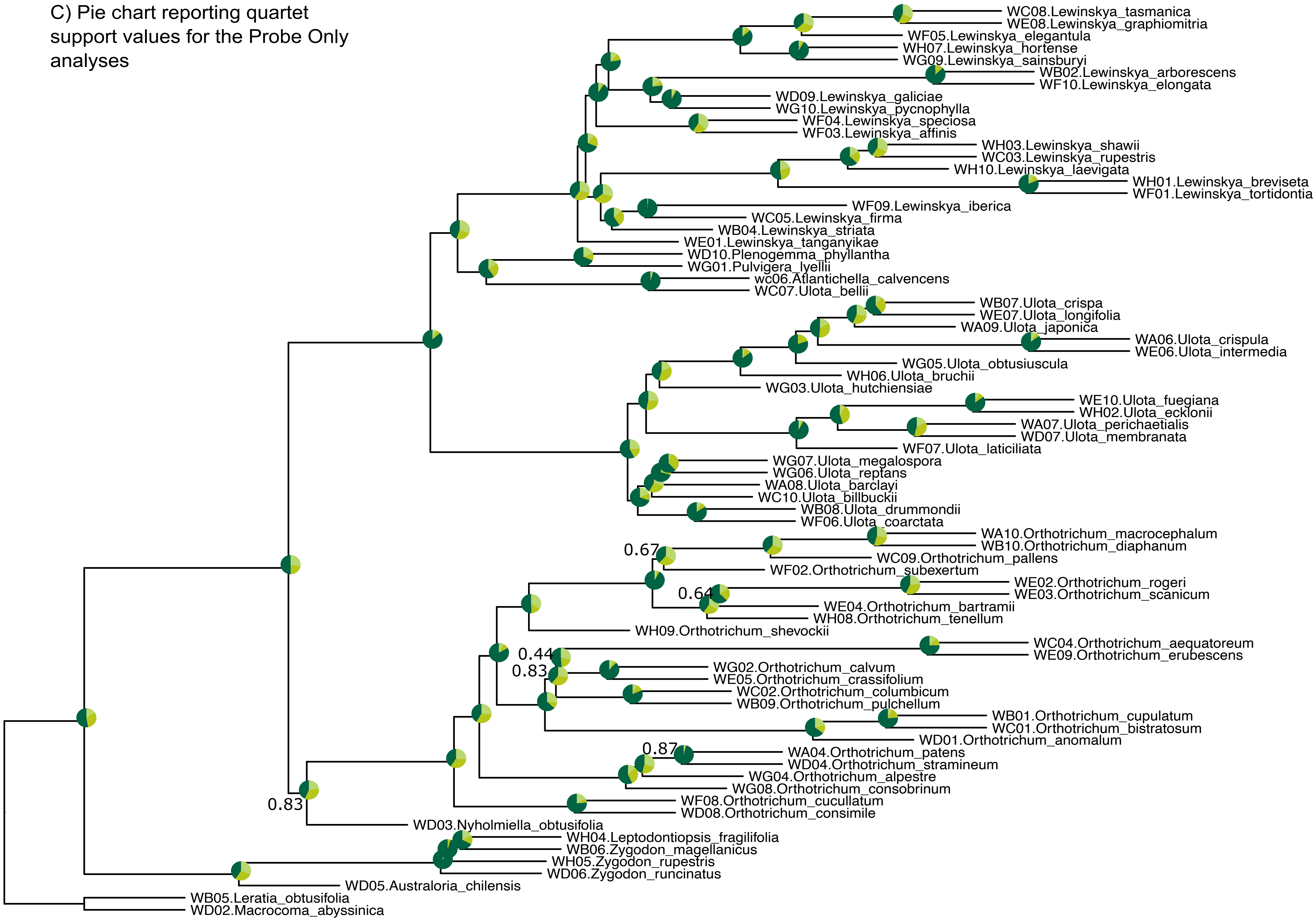

Supplement: Supplementary file 2 [file Data_Sheet_1.PDF]
